# Microwave Heating Promotes the S-Alkylation of Aziridine Catalyzed by Molecular Sieves: A Post-Synthetic Approach to Lanthionine-Containing Peptides

**DOI:** 10.3390/molecules26206135

**Published:** 2021-10-11

**Authors:** Valentina Verdoliva, Giuseppe Digilio, Michele Saviano, Stefania De Luca

**Affiliations:** 1 Institute of Biostructures and Bioimaging, National Research Council, 80134 Naples, Italy; valentina.verdoliva@gmail.com; 2 Department of Environmental, Biological and Pharmaceutical Sciences and Technologies, University of Campania “Luigi Vanvitelli”, Via Vivaldi 43, 81100 Caserta, Italy; 3 Department of Science and Technologic Innovation, Universitaà del Piemonte Orientale “A. Avogadro”, 15121 Alessandria, Italy; giuseppe.digilio@uniupo.it; 4 Institute of Crystallography, National Research Council, 70126 Bari, Italy; msaviano@unina.it

**Keywords:** microwave irradiation, zeolites, solid basic catalysis, post-synthetic modification, aziridine, lanthipeptide

## Abstract

Aziridine derivatives involved in nucleophilic ring-opening reactions have attracted great interest, since they allow the preparation of biologically active molecules. A chemoselective and mild procedure to convert a peptide cysteine residue into lanthionine via S-alkylation on aziridine substrates is presented in this paper. The procedure relies on a post-synthetic protocol promoted by molecular sieves to prepare lanthionine-containing peptides and is assisted by microwave irradiation. In addition, it represents a valuable alternative to the stepwise approach, in which the lanthionine precursor is incorporated into peptides as a building block.

## 1. Introduction

Aziridines have been recognized as valuable N-heterocyclic substrates, since these allow the preparation of versatile building blocks that are useful [1,2,3,4] for organic and medicinal chemistry applications. The aziridine ring-opening mechanism leads to the formation of unnatural amino acids, such as lanthionines. These are key components of lantibiotics, a peptide-based class of natural antibiotics able to overcome bacteria resistance issues through their peculiar action mechanism [5]. 

Recently, we reported on a synthetic strategy based on activated zeolites (4 Å molecular sieves). It allows the introduction of molecular diversities to peptide sequences via a nucleophilic substitution reaction promoted by the mild basic catalysis of 4 Å MS (molecular sieves) [6,7,8,9,10]. For several substrates introduced on peptide side chains, this protocol was further implemented by MW irradiation, which shortened the reaction time from hours to minutes [11].

MW heating has become an invaluable technology in organic synthesis. It is worth remembering that MW radiation is not only able to shorten the reaction time of chemical processes dramatically, but is also proven to reduce by-product formation, increase yield, and improve reproducibility. In this regard, MW heating is one of the most competitive technologies for the rapid optimization of reactions, as well as for discovering and probing the reactivity of new compounds. In addition, the issue of time saving is particularly relevant to the field of medicinal chemistry during lead compound generation [12,13,14,15,16,17]. The entry of MW radiation into pharmaceutical science is an invaluable additional tool for the rapid delivery of drug candidates. 

Our group has recently tuned a strategy to prepare lanthipeptides by using activated 4 Å MS as a catalyst (MS protocol). The reaction consists of a post-synthetic modification of fully unprotected peptide sequences, which can be performed via the S-alkylation of a cysteine inserted into a peptide sequence by cyclic sulfamidates. The obtained modified peptide contains a stereochemically pure lanthionine residue [18,19]. 

To investigate our procedure further, we decided to pursue the chemical synthesis of lanthipeptides by using a precursor other than cyclic sulfamidates. Namely, we applied this strategy to lanthipeptides via the nucleophilic ring-opening of aziridines upon the thiol attack of a cysteine residue catalyzed by MS. Given that our MS strategy employs MW technology, the design of the synthetic protocol includes MW irradiation as an additional parameter. 

## 2. Results and Discussion

In 2011, a protocol for the preparation of β-methyllanthionine by the nucleophilic attack of a cysteine on N-carboxyaziridines promoted by a Lewis acid was reported [20]. This protocol was characterized by a moderate yield. Another synthetic protocol for preparing protected β-MeLan as building blocks for the synthesis of the natural lanthibiotic Mersacidin was published recently [21]. This procedure uses InCl_3_ as the catalyst, a fully protected cysteine derivative, and an aziridine substrate activated toward sulfhydryl nucleophilic attack by a suitable electronegative substituent on the ring nitrogen. Both strategies are much more time-consuming than our approach of preparing lanthionine-containing peptides directly. In addition, the post-synthetic approach avoids generating steric hindrance problems with the building block during its coupling with the peptide on the resin and, as a consequence, requiring a large amount of it.

Herein, following our preferred strategy of directly introducing chemical diversities into peptide molecules under the basic catalysis of activated zeolites, we report on a selective synthetic strategy to introduce lanthionine derivatives within a linear peptide sequence. The final aim is to be able to generate a thioether bridge, which is the characteristic key structural motif of lanthibiotics.

The employed synthetic protocol relies on the S-alkylation of a cysteine by reaction with commercially available aziridines. A deep investigation was undertaken using different peptide models and the same (1,2-di-tert-butylaziridine 1,2-dicarboxylate) derivative. The general procedure used for the MW-assisted S-alkylation reaction promoted by molecular sieves was as follows. The crude peptides were placed into a reactor vessel and dissolved in DMF, a solvent that strongly adsorbs MW energy, under an argon atmosphere. Then, 1.2 equivalents of the same aziridine derivative and, as final step, activated 4 Å MS (T = 280 °C for 4 h under vacuum), previously cooled to room temperature, were also placed in the rector, which was quickly sealed. The reaction mixture was irradiated and stirred for 5 min at 40 °C in a microwave source apparatus [11].

**Scheme 1:**
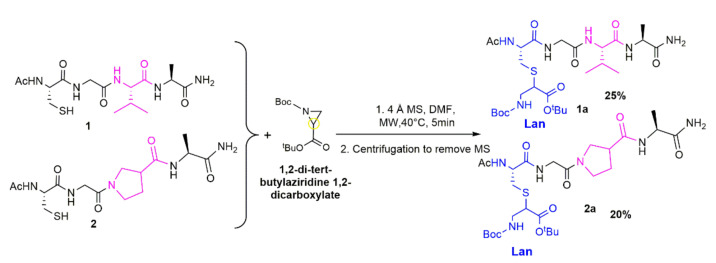
S-alkylation of Peptide 1 and 2 performed with 1,2-di-tert-butylaziridine 1,2-dicarboxylate.

Two model peptides (Val, Pro) were firstly alkylated in order to investigate the efficiency of the thiol substitution reaction promoted by MS on aziridine substrates (Scheme 1).

The yield was evaluated by the ratio between the HPLC peak area of the desired final product and the total area relative to the unreacted peptide and other by-products (Table 1).

**Scheme 2:**
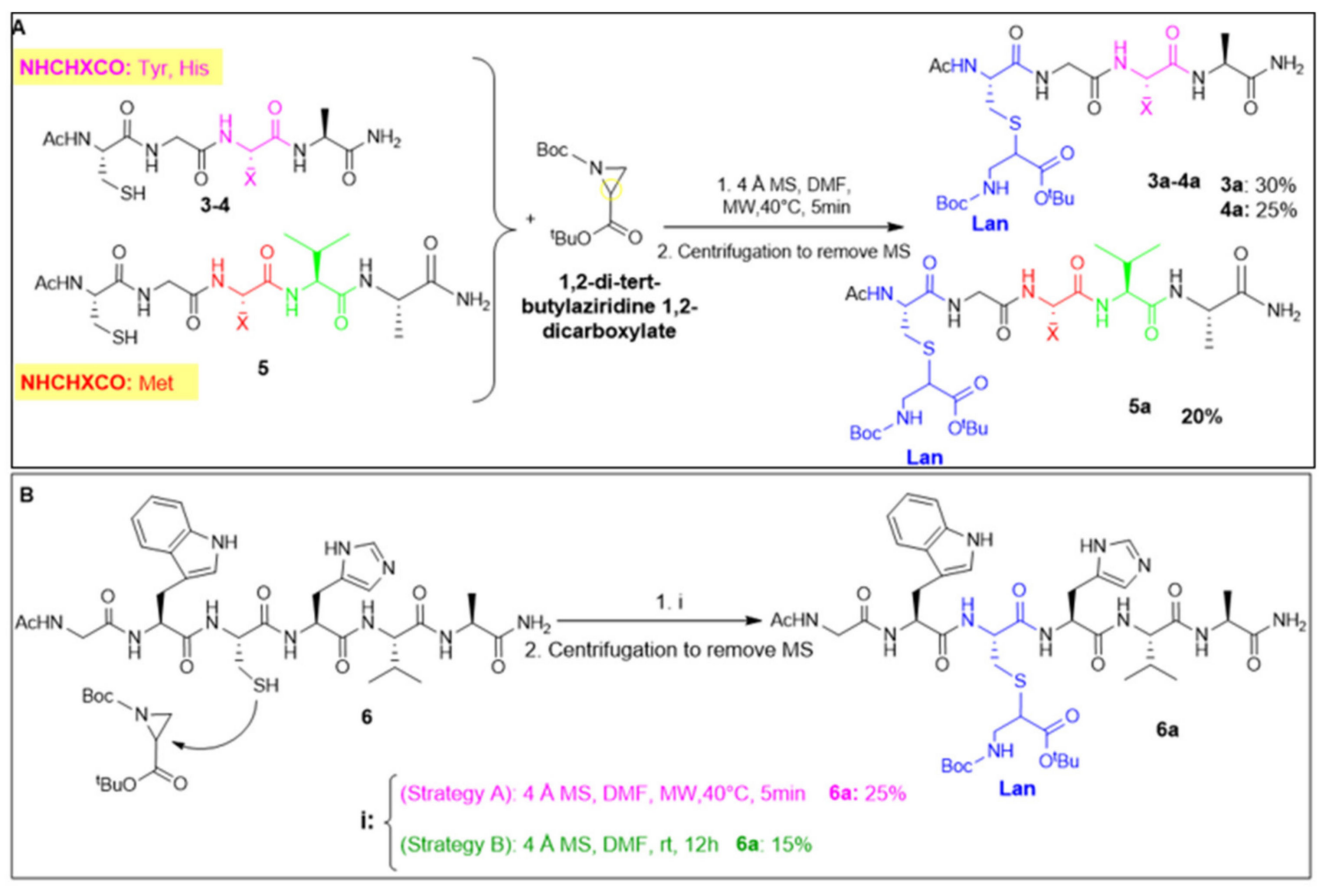
S-alkylation of peptide sequences performed with 1,2-di-tert-butylaziridine 1,2-dicarboxylate.

Next, we reacted different peptide sequences (Table 1) in order to assess whether our protocol of converting a cysteine in lanthionine by means of an aziridine precursor was chemoselective (Scheme 2).

**Figure 1:**
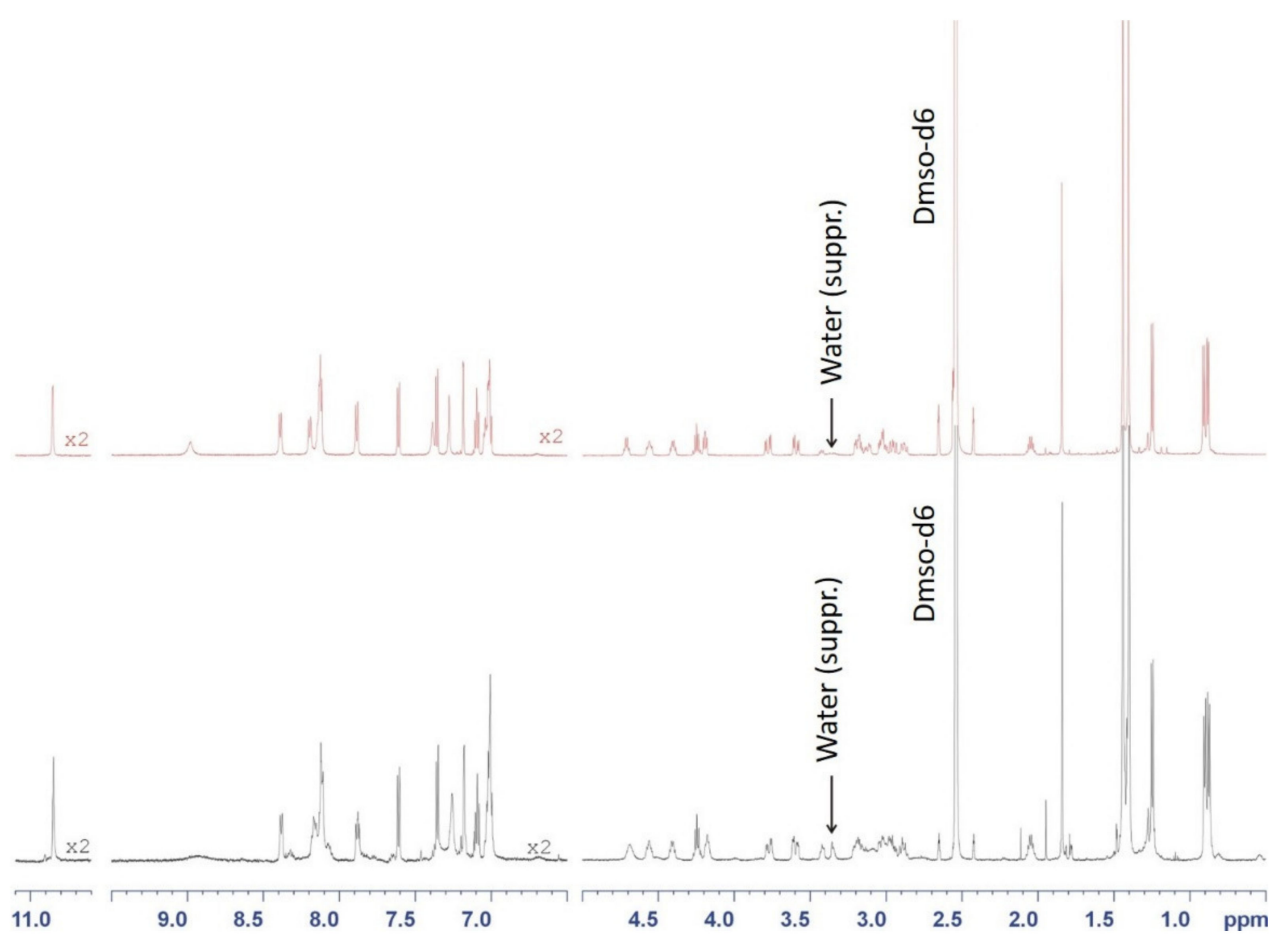
Superposition of the 1H-NMR spectra (dmso-d6, 300K) of compound **6a** as obtained from the aziridine precursor (black) and from the cyclic isoserine-sulfamidate precursor (compound **1d** in [19]).

Peptide **6** was designed to test chemoselectivity, as it contains a cysteine residue sandwiched in between potentially competing histidine and triptophan nucleophilic groups. In addition, the cysteine residue was in the middle of the peptide sequence, allowing us to investigate whether the different exposure level of the thiol cysteine group could affect its nucleophilicity and hence reaction yields. The reaction product was confirmed by NMR to correspond to the S-alkylation derivative. Product **6a** is the very same compound **1d** in [19], obtained by the cyclic isoserine-sulfamidate route of lanthipeptides. As a matter of fact, the 1H-NMR spectrum of compound **6a** (this work) was superimposable on that of compound **1d** [19] (Figure 1). 

**Figure 2:**
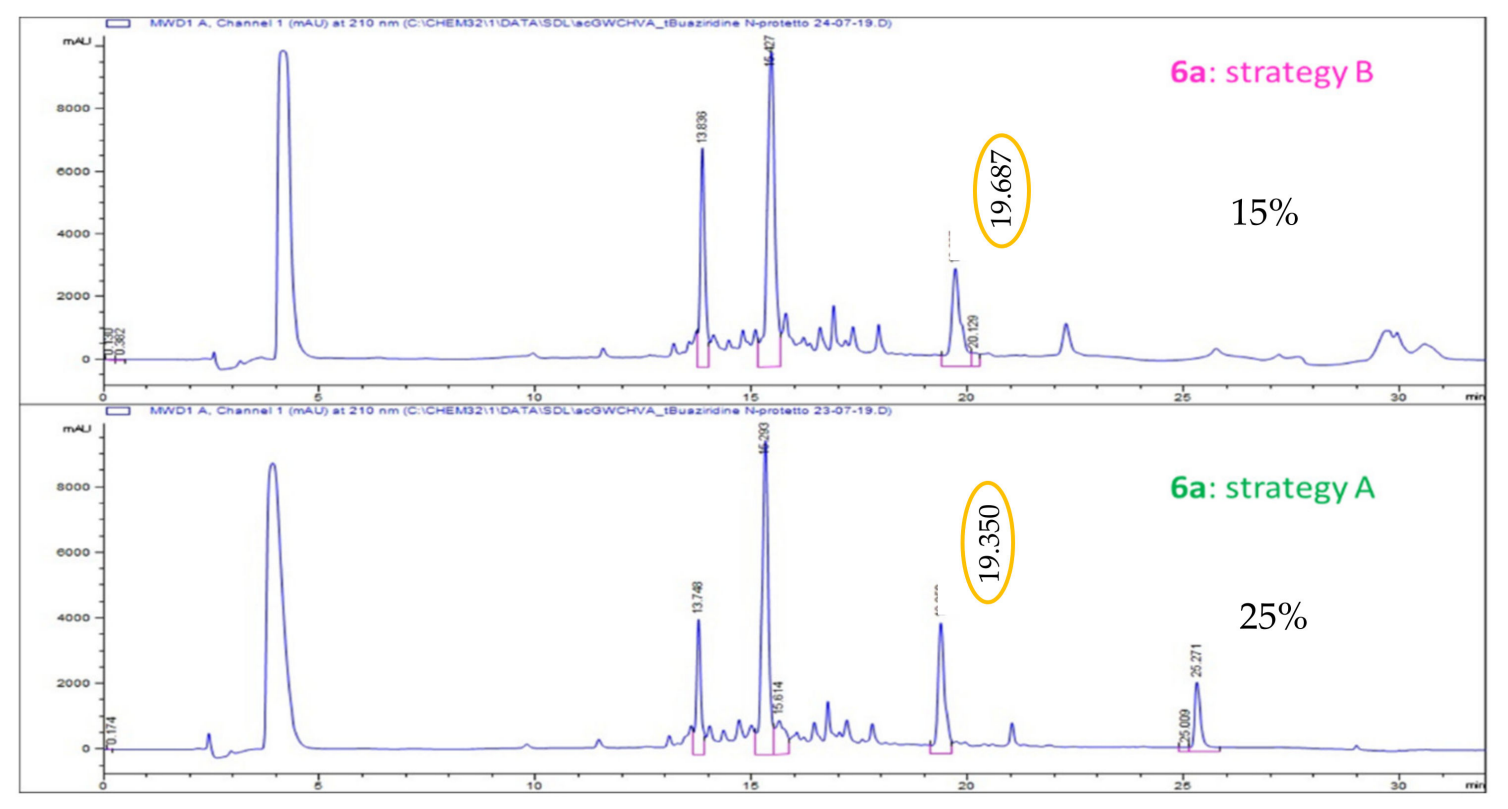
Superposition of the HPLC spectra of compound **6a** obtained from the aziridine precursor with strategy A (MW irradiation) and strategy B (T = rt).

The same reaction was performed following the MS protocol at room temperature (strategy B: 4 Å molecular sieves, t = 12 h under stirring, T = rt) in order to compare the yield of the final product obtained with and without MW assistance. As shown in Figure 2, following strategy A (MW irradiation) the S-alkylation occurred in a percentage higher than that observed for the same reaction performed with strategy B (T = rt). 

## 3. Materials and Methods

Fmoc protected amino acids, Rink Amide MBHA resin, N-hydroxybenzotriazole (HOBt), and benzotriazol-1-yl-oxy-tris-pyrrolidino-phosphonium (PyBOP) were purchased from Calbiochem-Novabiochem (Laufelfingen, Switzerland); piperidine and diisopropylethylamine (DIPEA) were purchased from Fluka (Milwaukee, WI, USA); all solvents were purchased from Aldrich (St. Louis, MI, USA) or Fluka (Milwaukee, WI, USA) and were used without further purification, unless otherwise stated. Molecular sieves of type 4 Å (beads, diameter 1.6 mm) were purchased from Aldrich (St. Louis, MI, USA) and activated by heating at 280 °C for 4 h under a vacuum. The 1,2-di-tert-butylaziridine-1,2-dicarboxylate was purchased from AKos GmbH ( 79540 Lörrach, Germany).

For all RP-HPLC procedures, the system solvent used was H_2_O 0.1 % TFA (A) and CH_3_CN 0.1 % TFA (B) (detection performed at 210 nm and 280 nm). Preparative RP-HPLC runs were carried out on the HP Agilent Series 1200 apparatus using a Phenomenex (Torrance, CA, USA) Gemini column (5 μm NX-C18 110 Å-150 mm × 21.2 mm, AXIATM) with a flow rate of 15 mL min^−1^ and a linear gradient from 5% to 70% B in 20 min. 

LC-ESI-TOF-MS analyses were performed with an Agilent 1290 Infinity LC System coupled to an Agilent 6230 TOF LC/MS System (Agilent Technologies, Cernusco sul Naviglio, Italy). The system solvent used was H_2_O 0.05 % TFA (A) and CH_3_CN 0.05 % TFA (B) (using a Phenomenex (Torrance, CA, USA)) Jupiter column (3 μm C18 300 Å-150 mm× 2.0 mm); linear gradient starting from 5 % to 70 % B in 20 min; detection performed at 210 nm and 280 nm).

NMR spectra were acquired with a Bruker. Avance spectrometer operating at 14 T (corresponding to a proton Larmor frequency of 600 MHz), equipped with an inverse Z-gradient 5 mm BBI probe. Temperature was set to 300.0 K and controlled within ±0.1 K by means of the BTO2000 VTU system. Samples were dissolved in 600 μL of DMSO-d6 (99.9 atom %). The residual solvent resonance at 2.54 ppm was used as a secondary reference for chemical shift calibration. Spectra were processed by the Bruker Topspin 4 software package( Ettlingen, Germany).

### 3.1. Peptide Synthesis

Peptide synthesis was carried out manually using the solid-phase method using the standard Fmoc-protecting group strategy. Appropriate Fmoc-amino acid derivatives (Fmoc-Ala-OH, Fmoc-Val-OH, Fmoc-Gly-OH, Fmoc-Cys(Trt)-OH, Fmoc-Met-OH, Fmoc-His(Trt)-OH, Fmoc-Trp(Boc)-OH, Fmoc-Pro-OH and Fmoc-Tyr-OH) were employed and a Rink Amide MBHA resin (0.7 mmol g^–1^ substitution; 50 µmol scale) was used as a solid support, as it releases peptides amidated at C-terminus upon acid treatment. All Fmoc-amino acids were activated by an in situ PyBop/HOBt//DIPEA activation procedure. Amino acid coupling steps were monitored by Kaiser test after 60 min coupling cycles. Fmoc-deprotection was performed with 20% piperidine in DMF for 5 + 10 min. Peptide N-terminus was acetylated by treatment with a mixture of acetic anhydride (4.7%) and pyridine (4%) in DMF for 10 min. The cleavage from the solid support and the simultaneous deprotection of all side chains were performed by suspending the fully protected compound-resins in TFA/H2O/TIS (97:2:1) for 3 h. The peptides were isolated by precipitation in cold diethyl ether and centrifuged to form a pellet.

### 3.2. General Procedure for the MW-Assisted Peptide S-Alkylation (Compound ***6a***)

**Figure 3:**
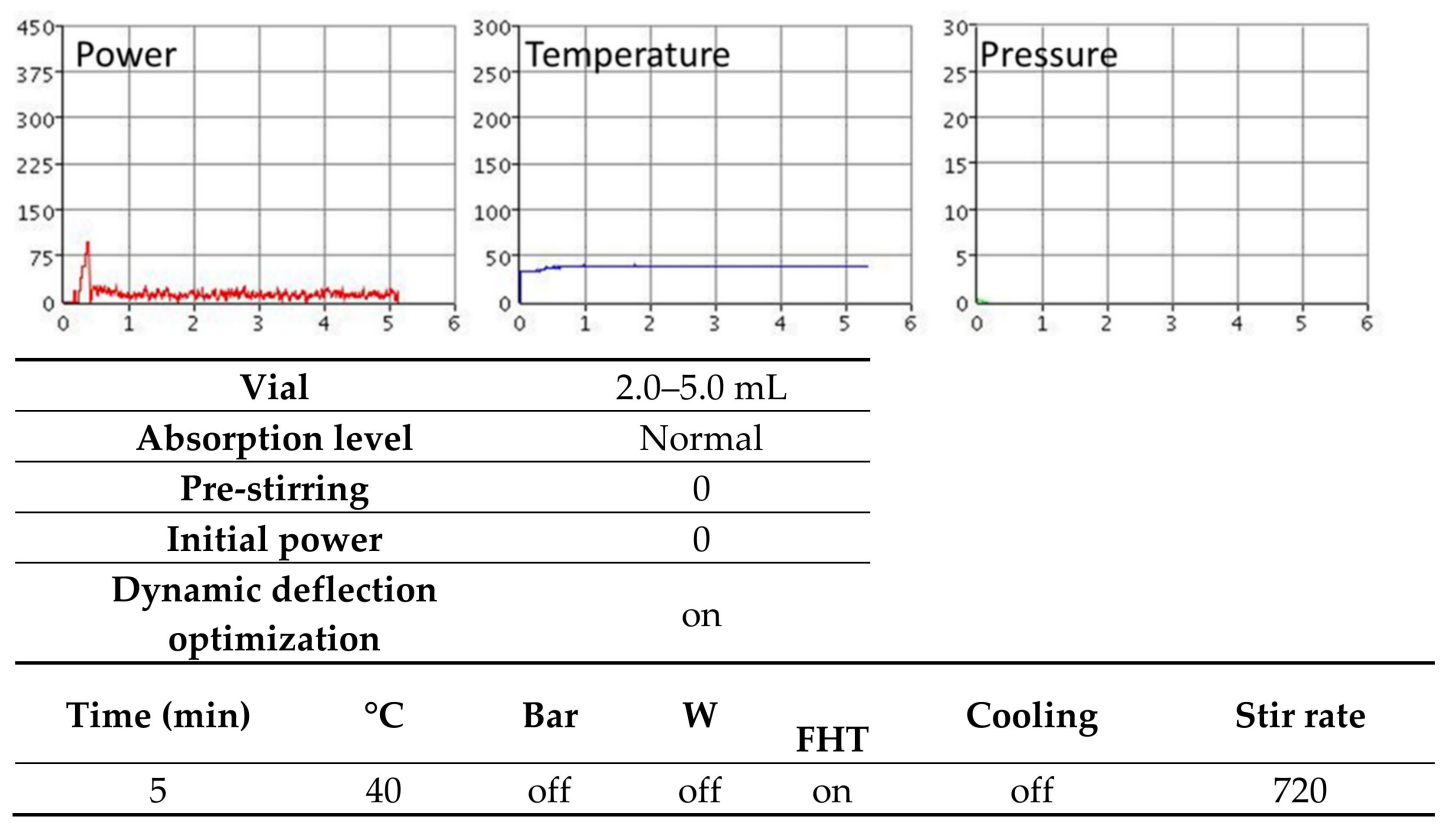
MW assisted S-alkylation of peptide **6a**.

A 4.7 mg mass of crude peptide 6 (0.0066 mmol) was dissolved in DMF (1.5 mL) under an argon atmosphere and placed in a 0.5–2 mL microwave vial. Then, 1,2-di-tert-butylaziridine-1,2-dicarboxylate (2 μL, 1.93 mg) was added by syringe. Lastly, 4 Å molecular sieves (1.5–2 g), previously activated at 280 °C for 4 h under vacuum (10^–4^ mBar), were placed into the microwave vial, which was quickly sealed. The obtained solution was irradiated for 5 min at 40 °C in a microwave oven (Initiator, Biotage Sweden AB, Uppsala, Sweden) and stirred at 720 rpm (Figure 3).

Direct temperature control was performed using infrared sensors. Then, the mixture was centrifuged to eliminate the sieves and the supernatant was concentrated under vacuum. The final product was purified by RP-HPLC, analyzed using mass spectrometry, and fully characterized by NMR spectroscopy.

Direct temperature control was performed using infrared sensors. Then, the mixture was centrifuged to eliminate the sieves and the supernatant was concentrated under vacuum. The final product was purified by RP-HPLC, analyzed using mass spectrometry, and fully characterized by NMR spectroscopy.

#### Compound **1a** strategy A. AcLanGlyValAlaNH_2_

A total of 5.8 mg of peptide 1 was reacted with 4.33 mg (4 µL) of 1,2-di-tert-butylaziridine-1,2-dicarboxylate.

Yield (**1a**) after RP-HPLC purification: 16% (0.9 mg); preparative HPLC t*R* = 12.929 min; ES-MS: calculated [M + H]*^+^*, 633.33, found *m*/*z* 633.3312 [M + H]*^+^*. White solid (see Supplementary Information).

#### Compound **2a** strategy A. AcLanGlyProAlaNH_2_

A total of 4.8 mg of peptide 2 was reacted with 3.6 mg (4 µL) of 1,2-di-tert-butylaziridine 1,2-dicarboxylate.

Yield (**2a**) after RP-HPLC purification: 14% (0.67 mg); preparative HPLC t*R* = 18.942 min; ES-MS: calculated [M + H]^+^, 631.30, found *m*/*z* 631.3131 [M + H]*^+^*. White solid (see Supplementary Information).

#### Compound **3a** strategy A. AcLanGlyTyrAlaNH_2_

A total of 5.0 mg of peptide 4 was reacted with 3.22 mg (3 µL) of 1,2-di-tert-butylaziridine 1,2-dicarboxylate. 

Yield (**4a**) after RP-HPLC purification: 21% (1.0 mg); preparative HPLC t*R* = 15.725 min; ES-MS: calculated [M + H]^+^ 697.32, found *m*/*z* 697.3198 [M + H]^+^. White solid (see Supplementary Information).

#### Compound **4a** strategy A. AcLanGlyHisAlaNH_2_

A total of 6.7 mg of peptide 3 was reacted with 4.58 mg (5 µL) of 1,2-di-tert-butylaziridine 1,2-dicarboxylate.

Yield (**3a**) after RP-HPLC purification: 18% (1.2 mg); preparative HPLC t*R*=19.245 min; ES-MS: calculated [M + H]*^+^* 671.32, found *m*/*z* 670.2782 [M + H]*^+^*. White solid (see Supplementary Information).

#### Compound **5a** strategy A. AcLanGlyMetValAlaNH_2_

A total of 5.0 mg of peptide 5 was reacted with 2.8 mg (3 µL) of 1,2-di-tert-butylaziridine 1,2-dicarboxylate.

Yield (**5a**) after RP-HPLC purification: 14% (0.7 mg); preparative HPLC t*R* = 12.929 min; ES-MS: calculated [M + H]^+^ 764.34, found *m*/*z* 764.3814 [M + H]^+^. White solid (see Supplementary Information).

#### Compound **6a** (Strategy A). AcGlyTrpLanHisValAlaNH_2_

Yield (**6a**) after RP-HPLC purification: 17% (0.8 mg); preparative HPLC t*R* = 19.350 min; ES-MS: calculated [M + H]^+^ 956.470, found *m*/*z* 956.4802 [M + H]^+^. White solid (see Supplementary Information).

#### Compound **1d** 1H-NMR

10.8 (s, 1H, Trp H_ε1_), 8.93 (s, br, 1H, His H_ε1_), 8.38 (d, 1H, Lan H_N_), 8.17–8.09 (overlap, 4H, His H_N_, Ala H_N_, Gly H_N_, Trp H_N_), 7.88 (d, 1H, Val H_N_), 7.61 (d, 1H, Trp H_ε3_), 7.36 (d, 1H, Trp H_ζ2_), 7.26 (br, 1H, CONH_2_), 7.18 (s, 1H, Trp H_δ1_), 7.09 (t, 1H, Trp H_η2_), 7.03–7.00 (overlap, 3H, Trp H_ζ3_, Lan H_ζ_, CONH_2_), 4.69 (m, 1H, His H_α_), 4.56 (m, 1H, Trp H_α_), 4.41 (m, 1H, Lan H_α_), 4.25 (m, 1H, Ala H_α_), 4.18 (m, 1H, Val H_α_), 3.77 (dd, 1H, Gly H_α1_), 3.60 (dd, 1H, Gly H_α2_), 3.42 (m, 1H, Lan H_α1_), 3.35 (m, overlapping with water, Lan H_ε1_), 3.20–2.90 (overlap, 7H, Lan H_β1_/H_β2_, Lan H_ε2_, Trp H_β1_/H_β2_, His H_β1_/H_β2_), 2.04 (m, 1H, Val H_β_), 1.84 (s, 3H, acetyl), 1.44 (s, 9H, Lan O^t^Bu), 1.40 (s, 9H, Lan Boc), 1.25 (d, 3H, Ala H_β_), 0.90 (d, 3H, Val H_γ1_), 0.88 (d, 3H, Val H_γ2_).

#### Compound **6a** (Strategy B). AcGlyTrpLanHisValAlaNH_2_

A total of 5.0 mg of peptide 6 was reacted with 2.08 mg (2 µL) of 1,2-di-tert-butylaziridine-1,2-dicarboxylate in the presence of activated molecular sieves (T= 280 °C for 4 h under vacuum) and under an argon atmosphere.

The reaction was stirred at room temperature for 12 h and followed by analytical RP-HPLC. The reaction mixture was separated from the molecular sieves by centrifugation and washed with DMF (0.200–0.500 mL). The final product was purified by RP-HPLC and analyzed by mass spectrometry.

Yield (**6a**) after RP-HPLC purification: 8% (0.4 mg); preparative HPLC t*R* = 19.687 min; ES-MS: calculated [M + H]^+^, 956.470, found *m*/*z* 956.4802 [M + H]^+^. White solid (see Supplementary Information).

## 4. Conclusions

In summary, we have developed an alternative post-synthetic protocol for preparing lanthionine containing peptides that uses microwave activation to promote the nucleophilic attack of a cysteine residue on aziridine substrates under the basic catalysis of zeolites (4 Å MS). The advantage of the aziridine route consists of its good stereochemical control [5], even though the reaction yield is moderate. Moreover, our strategy offers the valuable advantage of employing aziridine protected with acid-labile groups (Boc, t-Butyl), which are routinely used for peptide synthesis and are fully compliant with traditional protocols. Remarkably, we proved that the strong electron-withdrawing substituents on the aziridine nitrogen [18], which are generally required, can be avoided under our reaction conditions. In future, we plan to investigate the reactivity of N-unprotected aziridine [19] for the thiol substitution reaction in order to assess whether more flexible and versatile protocols can be developed to prepare lanthipeptides. The employment of MW radiation opens up opportunities to shorten the reaction time and increase the reaction yield to a certain extent too. Implementing MW heating as an additional parameter of the MS protocol can be considered as a valuable tool that speeds up the synthesis of modified peptides and has the potential to be employed as a lead compound in the pharmaceutic field.

## Figures and Tables

**Table 1:** Efficiency of the S-alkylation reaction to introduce lanthionine in peptides ^a^.

Entry	Peptide	Yield—MW ^b^ (%)	Yield—SC ^c^ (%)
**1a**	Ac**Lan**GlyValAlaNH_2_	25	-
**2a**	Ac**Lan**GlyProAlaNH_2_	20	-
**3a**	Ac**Lan**GlyTyrAlaNH_2_	30	-
**4a**	Ac**Lan**GlyHisAlaNH_2_	25	-
**5a**	Ac**Lan**GlyMetValAlaNH_2_	20	-
**6a**	AcGlyTrp**Lan**HisValAlaNH_2_	25	15

^a^ Legend: Lan = lanthionine with Boc and O^t^Bu protection. ^b^ MW condition: 5 min, 40 °C. ^c^ Standard condition: 12 h, room temperature.

## Data Availability

Not applicable.

## Supplementary Materials

The following are available online.
