# Supplementary material for: Microwave Heating Promotes the S-Alkylation of Aziridine Catalyzed by Molecular Sieves: A Post-Synthetic Approach to Lanthionine-Containing Peptides
Source: Molecules. 2021 Oct 11;26(20):6135. doi: 10.3390/molecules26206135 (PMC8538954; doi:10.3390/molecules26206135)
Supplement: Supplementary file 1 [file molecules-26-06135-s001.zip › molecules-1399501-supplementary.pdf]

Microwave heating promotes the S-Alkylation of Aziridine catalyzed by Molecular Sieves: post-synthetic approach to lanthionine-containing peptides

Valentina Verdoliva [1,4], Giuseppe Digilio [2], Michele Saviano [3], Stefania De Luca [1,*]

[1] *Institute of Biostructures and Bioimaging, National Research Council, 80134 Naples, Italy*

[2] *Department of Science and Technologic Innovation Università del Piemonte Orientale "A. Avogadro", 15121 Alessandria, Italy*

[3] *Institute of Crystallography, National Research Council, 70126 Bari, Italy*

[4] *Department of Environmental, Biological and Pharmaceutical Sciences and Technologies, University of Campania "Luigi Vanvitelli", Via Vivaldi 43, 81100 Caserta, Italy.*

Supporting Information

2. HPLC, MS, NMR Spectra S2

Compound 1a..............S2

Compound 2a..............S3

Compound 3a..............S4

Compound 4a..............S5

Compound 5a..............S6

Compound 6a..............S7

3. Abbreviations S9

Compound 1a strategy A

AcLanGlyValAlaNH$_2$

A. HPLC profile of the reaction crude product:

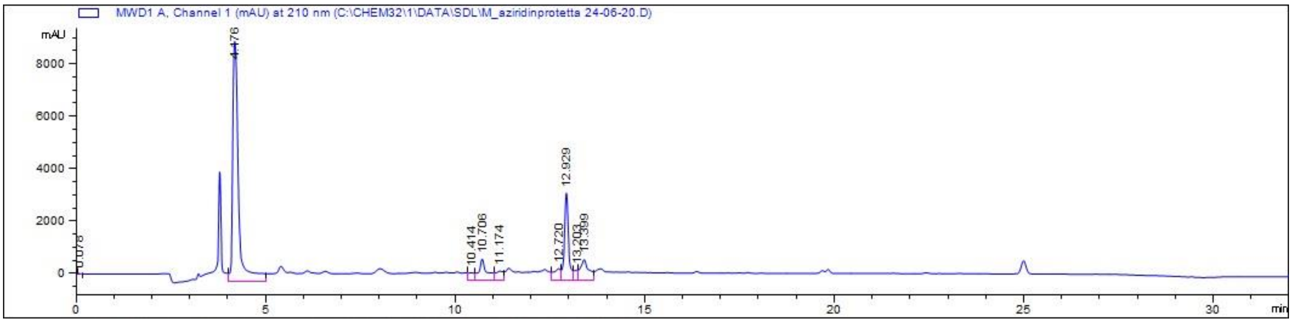

B. MS spectrum of **1a** after purification:

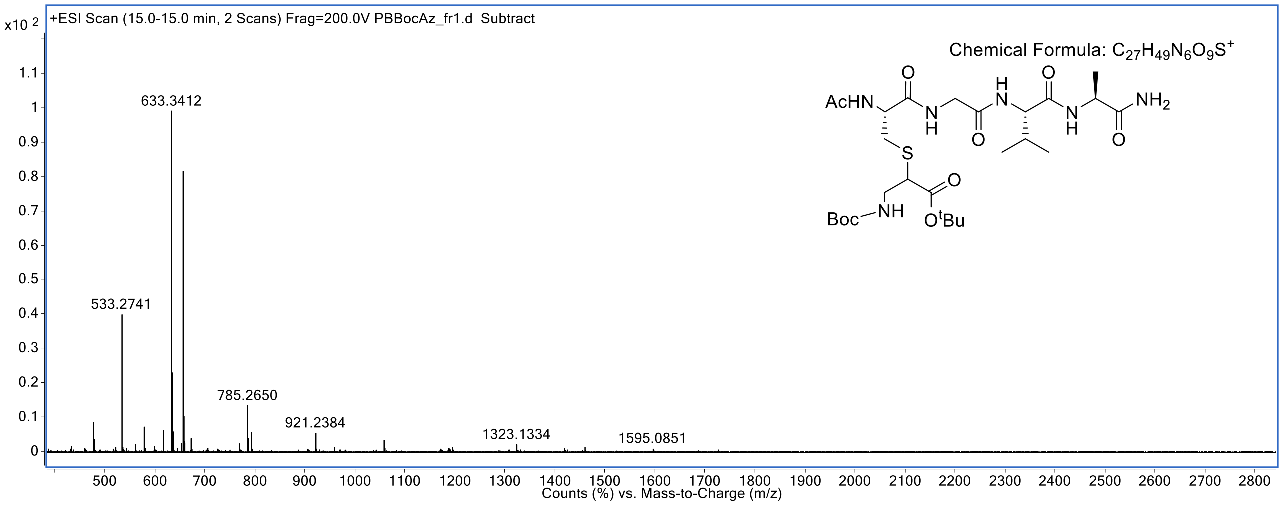

Compound 2a strategy A

AcLanGlyProAlaNH2

A. HPLC profile of the reaction crude product:

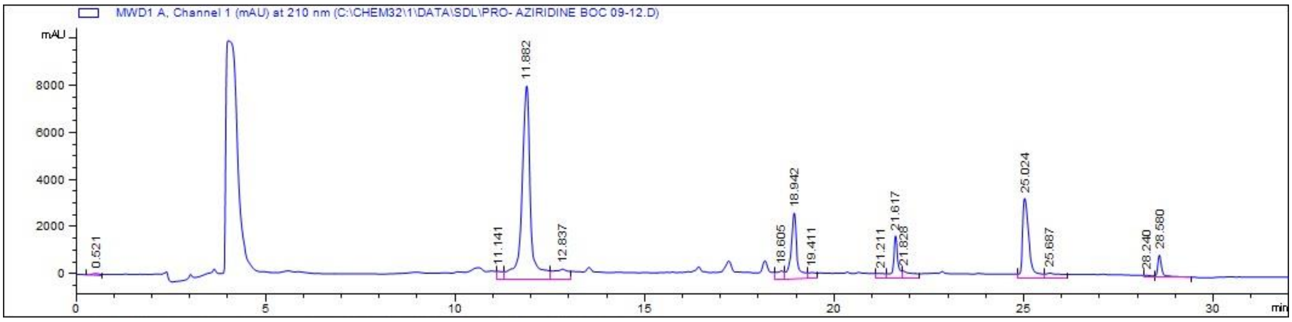

B. MS spectrum of **2a** after purification:

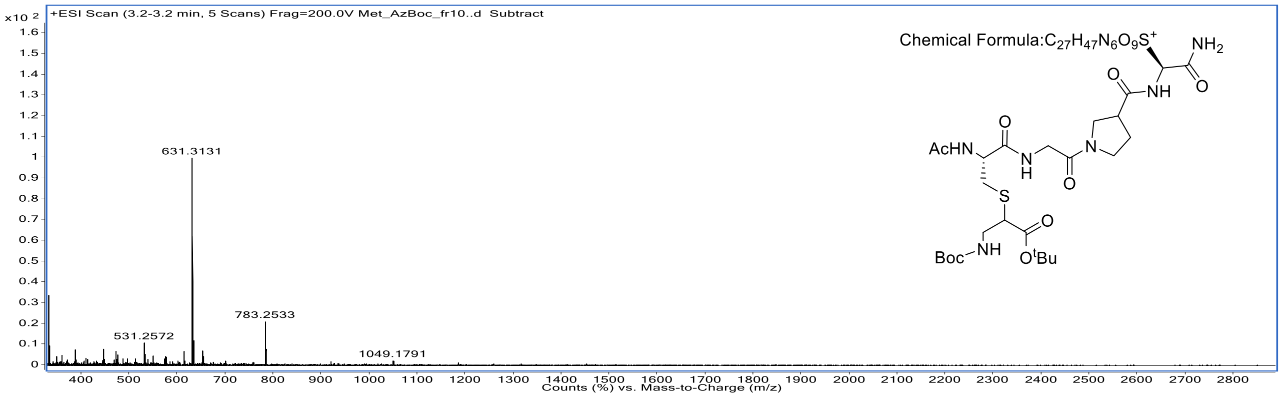

Compound 3a strategy A

AcLanGlyTyrAlaNH_2

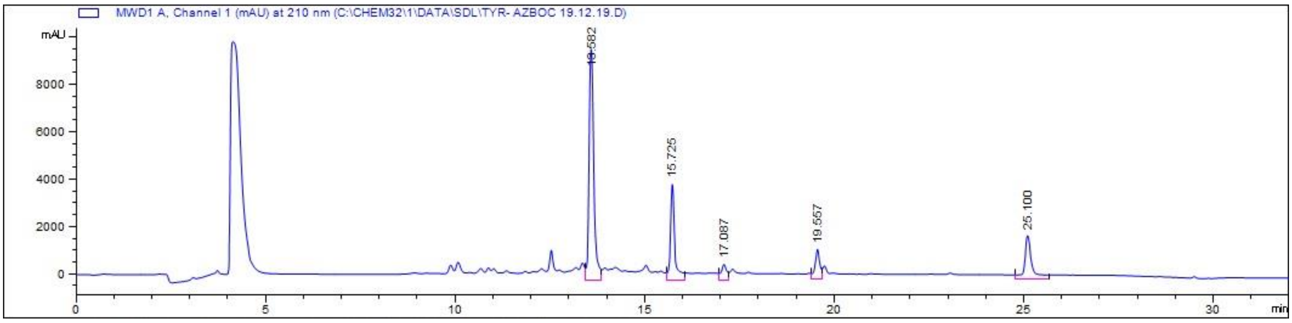

A. HPLC profile of the reaction crude product:

B. MS spectrum of **3a** after purification:

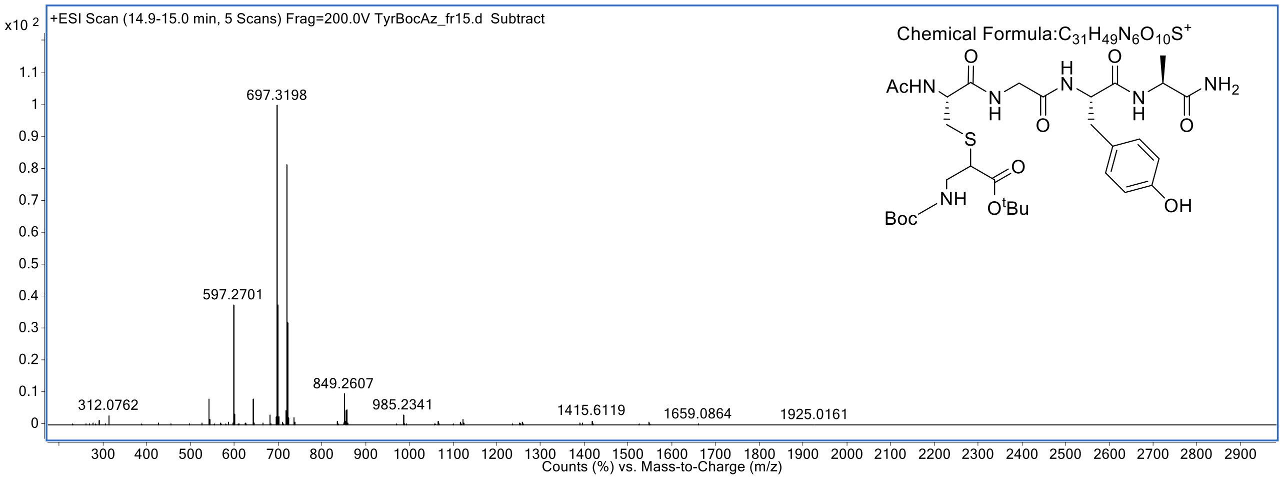

Compound 4a strategy A

AcLanGlyHisAlaNH$_2$

A. HPLC profile of the reaction crude product:

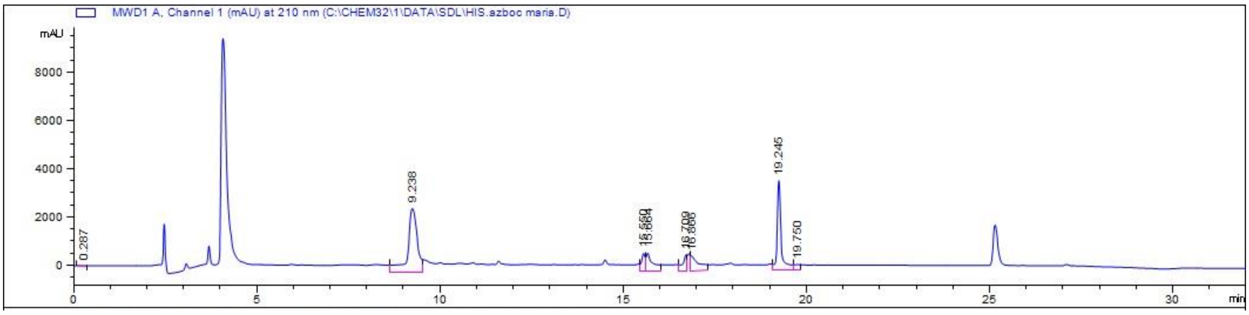

B. MS spectrum of **4a** after purification:

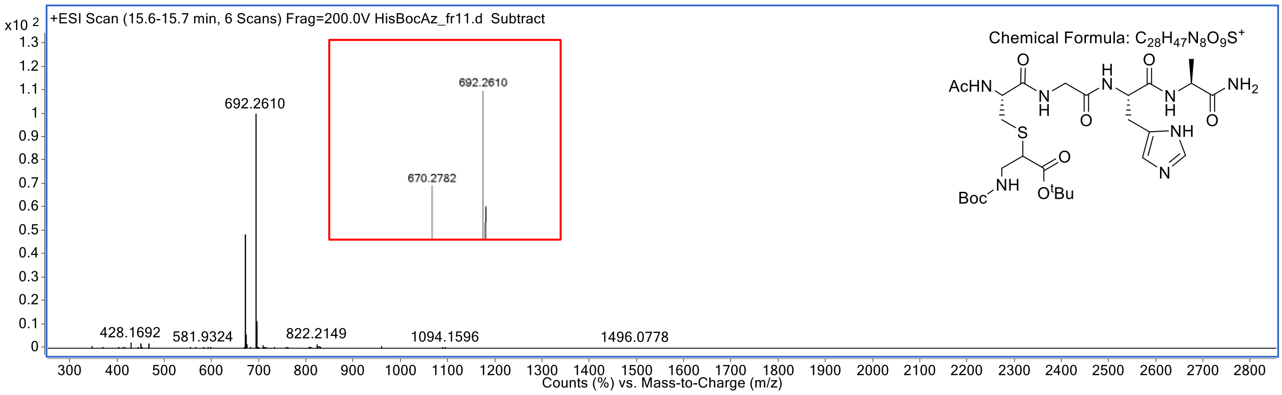

Compound 5a strategy A

AcLanGlyMetValAlaNH$_2$

A. HPLC profile of the reaction crude product:

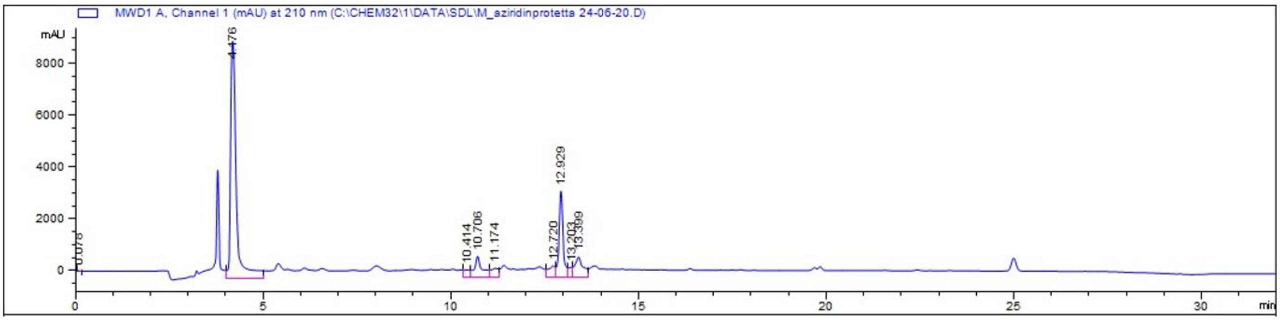

B. MS spectrum of **5a** after purification:

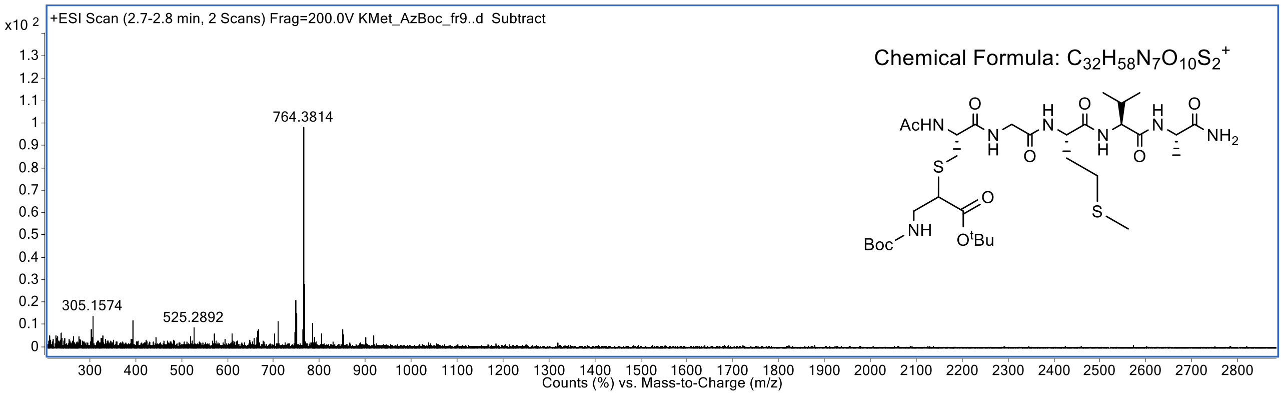

Compound 6a strategy A

AcGlyTrp**Lan**HisValAlaNH$_2$

A. HPLC profile of the reaction crude product:

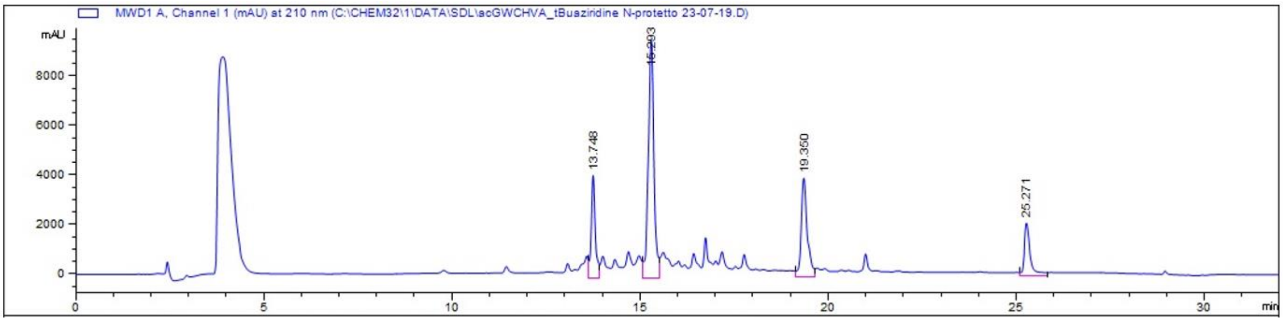

B. MS spectrum of **6a** after purification:

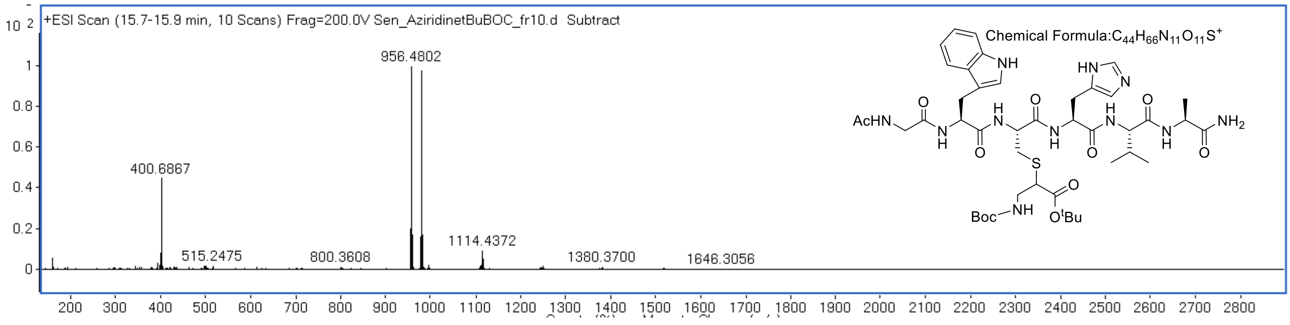

C. ^{1}H-NMR (600 MHz, dmso-d_6, 300 K).

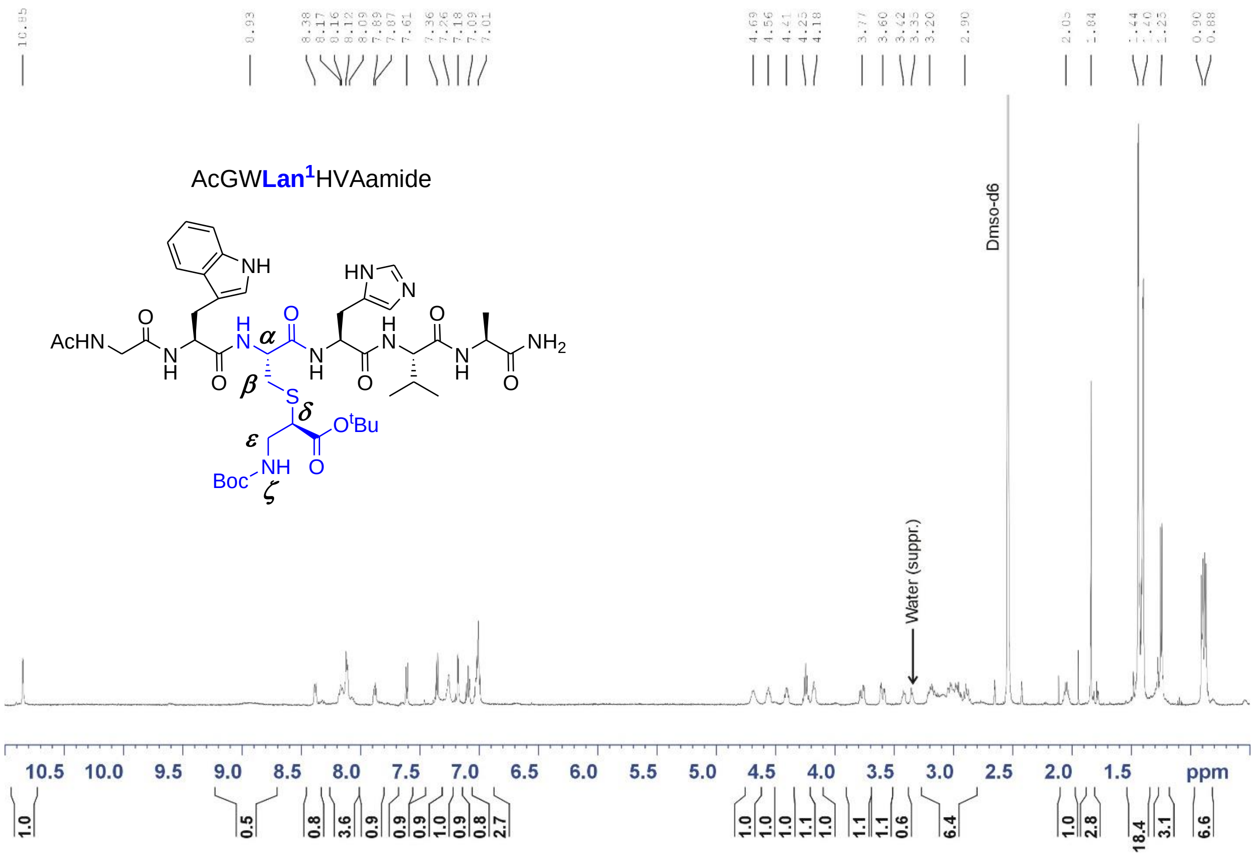

Compound 6a strategy B

AcGlyTrp**Lan**HisValAlaNH_2

A. HPLC profile of the reaction crude product:

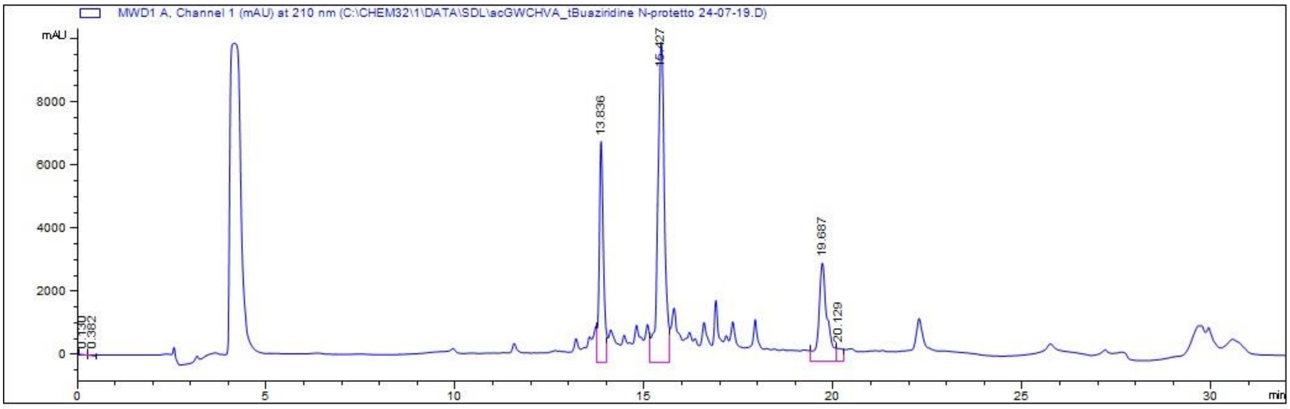

Abbreviations

Boc: *Tert*-butoxycarbonyl

DBU:1,8-diazabyciclo[5.4.0]undec-7-ene

DCM: Dichloromethane

DIPEA: Diisopropylethylamine

DMF: *N*,*N*-Dimethylformamide

DMSO-d_6: Dimethylsulfoxide-d_6

Fmoc: 9-Fluorenylmethoxycarbonyl

HOBt: *N*-hydroxybenzotriazole

PyBOP: Benzotriazol-1-yl-oxy-tris-pyrrolidino-phosphonium

^{t}Bu: *Tert*-butyl

TFA: Trifluoroacetic acid

TEA:Triethylamine

TIS:Triisopropylsilane
